# Transforming growth factor-β and integrins: key players in EMT and breast cancer progression

**DOI:** 10.1186/s12935-026-04196-4

**Published:** 2026-02-11

**Authors:** Prasanna Srinivasan Ramalingam, Muhammad Afzal, Manjunath Mirle Rekha, Samir Sahoo, Surya Nath Pandey, Chandana Maji, Kavita Goyal, Haider Ali, Sachin Kumar Singh, Gaurav Gupta, Md Sadique Hussain, Janaki Ramaiah Mekala, Sivakumar Arumugam

**Affiliations:** 1 Protein Engineering Lab, School of Biosciences and Technology, Vellore Institute of Technology, Katpadi, Vellore, 632014 Tamil Nadu India; 2 Department of Pharmaceutical Sciences, Pharmacy Program, Batterjee Medical College, Jeddah, 21442 Saudi Arabia; 3 Department of Chemistry and Biochemistry, School of Sciences, JAIN (Deemed to Be University), Bengaluru, Karnataka India; 4 Department of General Medicine, IMS and SUM Hospital, Siksha ‘O’ Anusandhan (Deemed to be University), Bhubaneswar, Odisha India; 5 Department of Pharmacology, Teerthanker Mahaveer College of Pharmacy, Teerthanker Mahaveer University, Moradabad, Uttar Pradesh India; 6 Noida Institute of Engineering and Technology (Pharmacy Institute), 19 Knowledge Park 2, Greater Noida, India; 7 Sharda University, Greater Noida, Uttar Pradesh India; 8 Department of Pharmacology, Kyrgyz State Medical College, Bishkek, Kyrgyzstan; 9 School of Pharmaceutical Sciences, Lovely Professional University, Phagwara, 144411 India; 10 Centre for Research Impact and Outcome, Chitkara College of Pharmacy, Chitkara University, Rajpura, Punjab India; 11 Uttaranchal Institute of Pharmaceutical Sciences, Uttaranchal University, Dehradun, 248007 Uttarakhand India; 12 School of Biosciences and Technology, Vellore Institute of Technology, Katpadi, Vellore, 632014 Tamil Nadu India

**Keywords:** Transforming growth factor-β, Breast cancer, Invasion, Metastasis, TME, EMT

## Abstract

In advanced breast cancer, the integrin-TGF-β axis moves from a tumor-suppressive to a pro-metastatic state and is central to breast cancer metastasis. We review how integrins, especially αvβ3, αvβ6, and β1, serve as critical modulators of TGF-β signaling and play a key role in epithelial-to-mesenchymal transition (EMT), immune evasion, angiogenesis, and ECM remodeling. In addition to activating latent TGFβ, these integrins also function as downstream effectors that fuel EMT and tumor progression through a feedback loop. Integrins function mechanistically to promote cytoskeletal reorganization, ECM degradation, and tumor cell motility, and TGF-β signaling influences integrin expression and activity. Targeting this axis is explored as a therapeutic approach to reduce metastasis and improve patient outcomes, and the potential of integrin inhibitors, TGFβ pathway blockers, and combination therapies are discussed. This review highlights the need for novel interventions to disrupt this axis by elucidating the interplay between integrins and TGF-β.

**Graphical Abstract:**

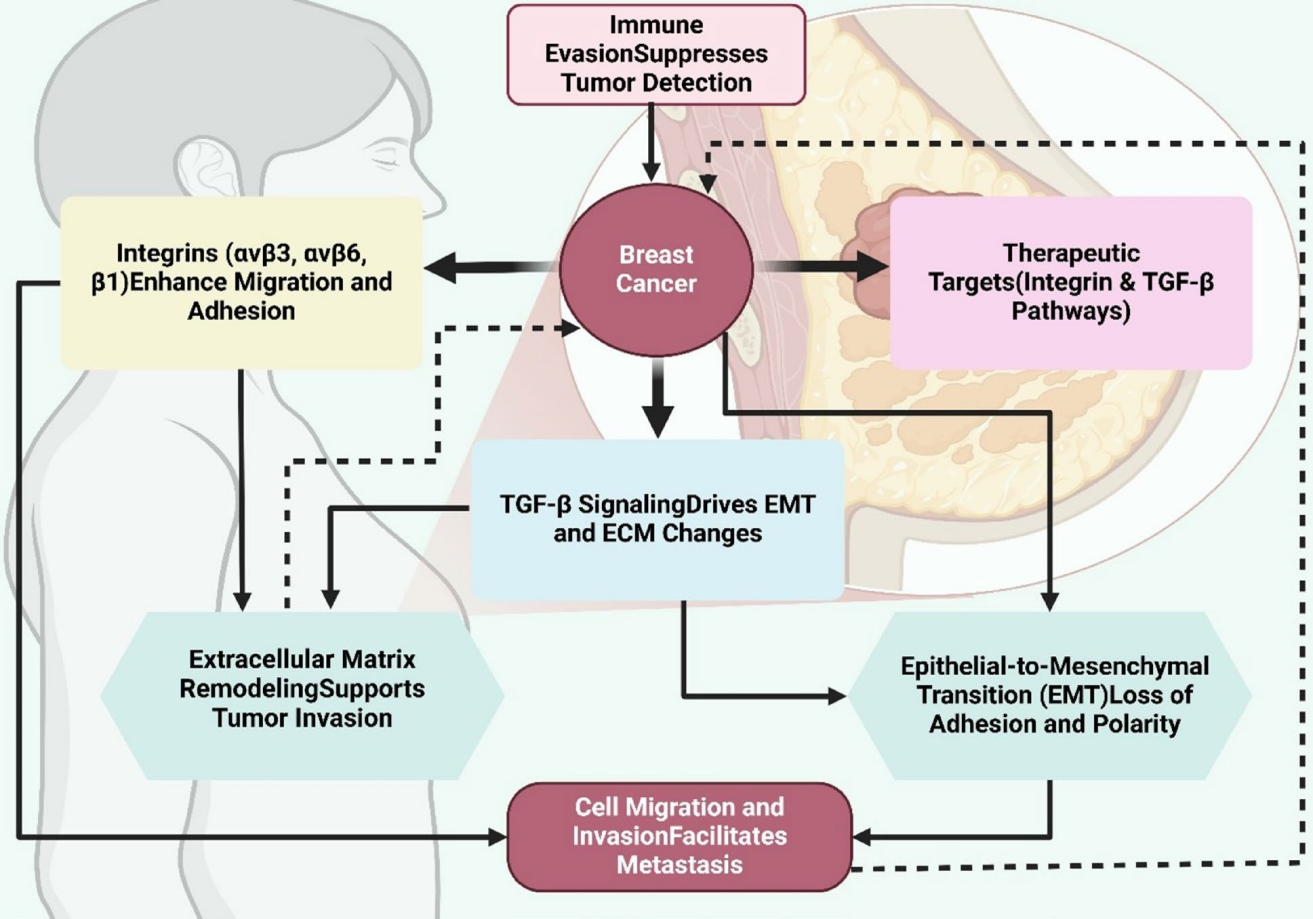

## Introduction

Metastasis continues to represent the primary cause of mortality in breast cancer patients worldwide; breast cancer continues to be a predominant cause of cancer-related mortality in women [1]. Diagnosis and localized treatments have improved survival rates, but metastatic breast cancer remains a challenge. Metastasis is the dissemination of cancer cells from primary cancer to more than one ‘distant’ organ, including the lungs, the liver, the bones, or the brain, where secondary tumors develop. The local invasion, intravasation, retention in circulation, extravasation, and metastatic colonization in distant tissues is a complex, multi-step process [2, 3]. Intricate molecular interactions orchestrate each stage of metastasis; thus, metastasis is a highly dynamic and difficult to understand and therapeutically target process [4, 5]. TGF-β is a multifunctional cytokine that mediates many cellular processes: cell growth, differentiation, apoptosis, and immune responses [6]. In normal epithelial cells and at early stages of cancer, TGF-β functions as a tumor suppressor by inducing cell cycle arrest and apoptosis. During cancer progression, TGF-β genetic and epigenetic alterations in tumor cells allow their role from tumor suppressor to tumor promoter. Its ability to induce EMT is particularly highlighted following later stages of cancer advancement, where TGF-β switches from a cancer-inhibitory to a pro-oncogenic factor and drives loss of epithelial polarity, decrease of E-cadherin, and acquisition of a motile mesenchymal phenotype, thereby facilitating invasion and metastasis [7].

EMT is a biological process in which epithelial cells lose apical-basal polarity and cell-cell adhesion, and acquire mesenchymal characteristics, including increased motility and invasiveness [8]. TGF-β is a major inducer of EMT in breast cancer, resulting in tumor cells adopting a more aggressive phenotype and invasion into surrounding tissues for dissemination to distant organs [9, 10]. However, the expression of many key EMT markers can be regulated by TGF-β through Smad-dependent and independent pathways by modulating the expression of the epithelial marker genes, E-cadherin (CDH1), and the mesenchymal markers genes, N-cadherin (CDH2) and vimentin (VIM) [11]. Alterations in tight junction proteins accompany E-cadherin loss during EMT. Comparative analyses in invasive micropapillary carcinoma shown prognostic associations between claudin expression patterns and survival outcomes [12]. This remodeling of the cytoskeleton and cell migration takes place through these changes and allows cancer cells to enter the circulation by passing through the extracellular matrix (ECM).

Integrins are transmembrane glycoproteins that act as cell adhesion receptors and mediate intercalations between cells and the ECM. They are heterodimers of an α subunit and a β subunit, where each combination confers the specificity to specific ECM components of FN, collagen, or laminin [13]. The sensing and transduction of extracellular signals to the intracellular cytoskeleton and their effects on cell adhesion, migration, survival, and proliferation depend critically on integrins [14]. Integrins, especially αvβ3, αvβ6, and β1, are greatly upregulated and strongly involved in metastasis in breast cancer. These integrins drive EMT in activating downstream pathways, like MAPK, PI3K/ AKT, and FAK, which coordinately organize cytoskeletal reorganization and cell motility [15]. Moreover, integrins also facilitate ECM remodeling through interaction with matrix metalloproteinases (MMPs) and induction of their expression, as well as tumor invasion and dissemination [16].

Integrin-TGF-β interactions are critical for EMT and metastasis in breast cancer. TGF-β folded in the ECM with its proinflammatory cytokine receptor latent TGF-β binding protein (LTBP) and its latency-associated peptide (LAP) is regulated by integrins that mediate activation of stored latent TGF-β [17, 18]. To illustrate, αvβ6 integrin binds LAP to activate TGF-β and then induces EMT and associated invasive phenotypes. Moreover, TGF-β signaling affects integrin expression and function through a loop of reverse signaling that amplifies its tumor-promoting effects, and downstream integrin-activated pathways interconnect with TGF-β signaling elements. Specifically, integrin activation of FAK and Src facilitates TGF-β receptor phosphorylation for both Smad (and FKHR, for example) dependent and Smad (and FKHR, for example) independent signaling, e.g., p38MAPK and Rho/ROCK pathways. Together, these interactions drive EMT and immune evasion, which all lead to metastatic colonization. This synergy is exemplified by the αvβ3 integrin-TGF-β receptor axis that mediates invasion and subsequent secondary tumor establishment at distant organs. This review aims to elucidate the integrin-TGF-β axis in breast cancer EMT and metastasis, emphasizing its mechanistic intricacies and therapeutic potential. By shedding light on this critical interaction, we hope to pave the way for novel, targeted therapies to combat metastatic breast cancer, ultimately improving patient outcomes.

Despite some TGF-β independent effects of integrins in tumour progression, in this review we are mainly focusing on integrin activities that are mechanistically related to TGF-β signaling, especially where direct physical and functional crosstalk between the different pathways induces EMT and metastasis.

## TGF-β signaling in EMT

EMT, critical for cancer progression and metastasis, involves regulation of TGF-β signaling [19]. The canonical TGF-β pathway Smad dependent: TGF-β ligands bind to activate TβR-II that subsequently recruits and phosphorylates the TβR-I, leading to phosphorylation of receptor-regulated Smads (Smad2 and Smad3) [20]. Phosphorylated Smad2 and Smad3 form complexes with Smad4 that translocate into the nucleus, where they regulate the expression of EMT-related genes. The resulting in epithelial marker suppression, e.g., CDH1, and upregulation of mesenchymal markers, e.g., VIM, FN, and CDH2. Beyond Smad-dependent pathways, TGF-β activates non-canonical pathways, namely the PI3K/AKT, MAPK, and Rho GTPases pathways, as platforms for complementary and sometimes independent EMT drivers [21]. The PI3K/AKT pathway promotes cell survival and cytoskeletal reorganizing, while the MAPK pathway comprising ERK, JNK, and p38 leads to transcriptional transdifferentiation known to characterize EMT [22]. Migration and invasion behaviors are controlled by Rho-GTPase, including RhoA, Rac1, and Cdc42, which regulate cytoskeletal dynamics and cell polarity [23]. These pathways converge to orchestrate these cellular changes, including loss of apical-basal polarity and loss of cell-cell junction dissolution of motility. TGF-β mediated EMT proceeds through distinct transition phases, beginning with an early epithelial morphological change that includes downregulation of adhesion molecules such as CDH1 [24]. Intermediate stages are characterized by movement towards cytoskeletal reorganization, partial loss of epithelial characteristics, and upregulation of mesenchymal markers. This is the final phase, in which the tumor cells have a truly mesenchymal phenotype that allows tumor cells to detach, invade, and disperse. The impact of these changes on metastasis is not only critical, but they also confer stem cell-like properties and drug resistance, essential for tumor heterogeneity and aggressiveness [25, 26]. Therefore, TGF-β signaling through the canonical and noncanonical pathways represents a master regulator of EMT and represents a key therapeutic target in metastatic breast cancer.

## Integrins in EMT and cancer progression

### Structure and function of integrins

**Fig. 1:**
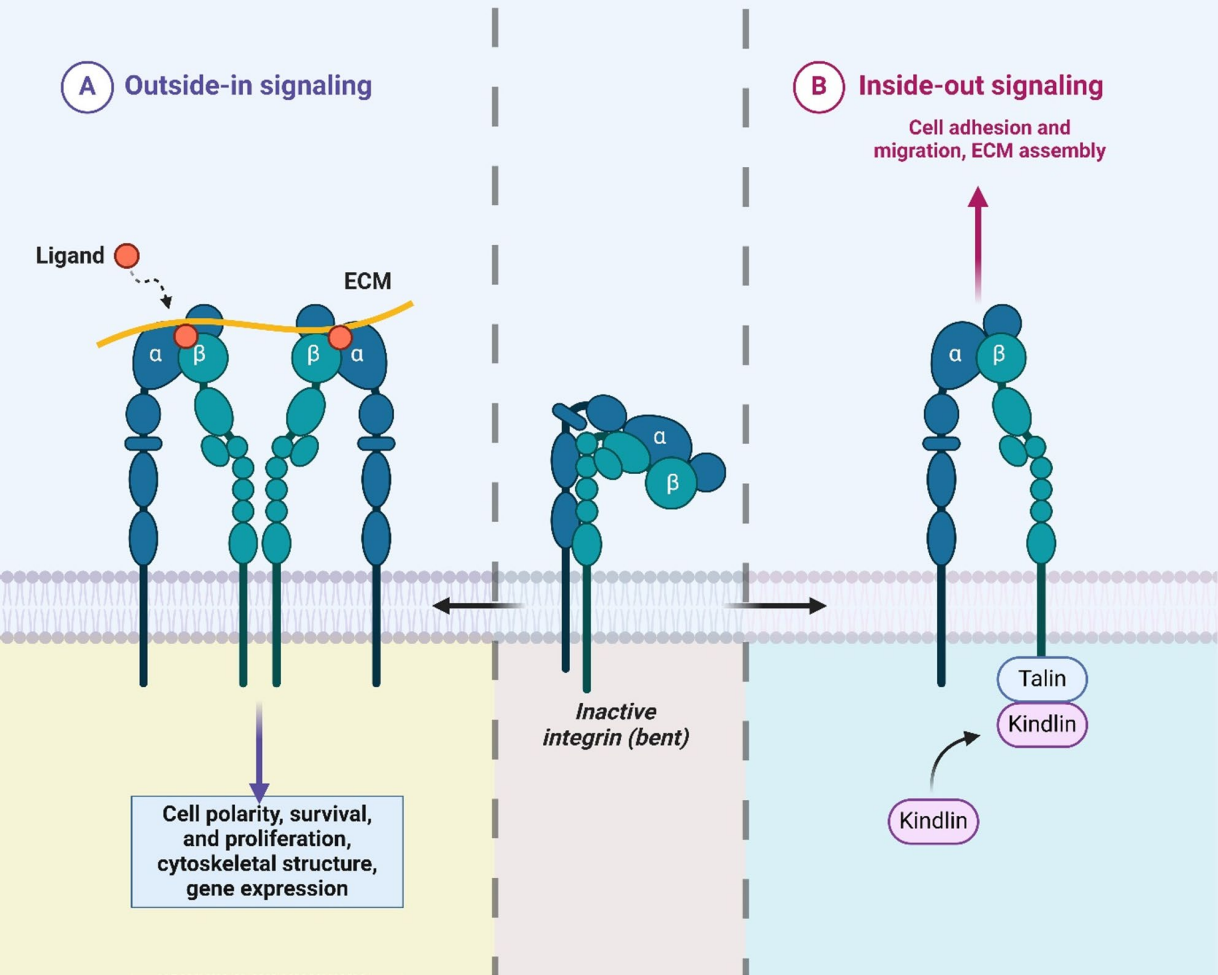
The figure demonstrates the dual functionality of integrins in mediating “outside-in” and “inside-out” signaling, essential for cellular communication and regulation. In outside-in signaling, integrins are activated by extracellular ligands binding to their α and β subunits, often through interaction with the extracellular matrix (ECM). This leads to intracellular responses that regulate cell polarity, survival, proliferation, cytoskeletal organization, and gene expression. In contrast, inside-out signaling begins with intracellular adaptor proteins, such as Talin and Kindlin, binding to the integrin cytoplasmic tails, transitioning integrins from an inactive state to an active conformation

Integrins are heterodimeric transmembrane receptors composed of one α and one β subunit, forming more than 24 distinct combinations in mammals [27]. Different heterodimer ligand binding properties allow each heterodimer to interact with the components of the ECM, such as collagen, laminin, and FN [28]. The cell adhesion, migration, survival, and signaling events facilitated by these interactions are central to tissue homeostasis and cancer progression. Bidirectional integrin signaling is mediated by integrins acting both as receptors and transmitters of external signals [29]. Integrins’ binding to ECM ligands leads to outside-in signaling, regulated by intracellular signaling cascades, which control cytoskeletal reorganization, cell polarity, and motility [30]. “Inside out” signaling, on the other hand, is extracellular activators such as talin and kindlin, which in turn trigger changes in integrin conformation and thereby increase their affinity to ECM ligands [31]. By establishing integrin as critically involved in processes such as wound healing, immune responses, and cancer progression, this bidirectional signaling has been established [32]. Integrins function as molecular bridges that coordinate cell-ECM interactions and help to transmit external mechanical cues into intracellular signaling networks, acting as key regulators of EMT and metastatic behavior in cancer cells [33]. A schematic overview of integrin bidirectional (‘outside-in’ and ‘inside-out’) signalling and its relevance to EMT and TGF-β crosstalk is provided in Fig. 1.

### Integrins in EMT

Central to TGF-β activation, a key step in EMT, integrins potentiate TGF-β activity, including αvβ6 and αvβ8, and α5β1 [34]. The integrins αvβ8 and αvβ6 interact with the LAP of TGF-β, facilitating its activation, as well as induction of EMT; αvβ6 integrin binds LAP–TGF-β complexes and promotes activation under mechanical tension, and αvβ8 integrin activates TGF-β via proteolytic cleavage. α5β1 integrin also enhances TGFβ signaling by modulating the spatial organization of the receptor [35]. Integrins also play a role in driving cytoskeletal reorganization needed for EMT downstream of TGF-β activation. Integrins harness actin cytoskeleton dynamics by regulating FAK and ILK signaling pathways through which cell shape changes, motility, and breakdown of cell-cell junction are regulated [36]. Collectively, these mechanisms provide tumor cells the ability to acquire invasive properties and facilitate metastasis [37].

### Integrin dysregulation in breast cancer

Breast cancer progression is characterized by Integrin dysregulation [38]. Specific integrins, such as αvβ3, αvβ6, and β1 integrins, are overexpressed in aggressive breast carcinoma subtypes and correlated to poor prognosis [39]. Integrin levels in normal and tumor cells are elevated, and upon activation, they further enhance tumor cell adhesion, migration, and invasion through activation of FAK, MAPK, and PI3K/AKT signaling pathways. αvβ6 integrin up-regulates the expression of TGF-β and promotes EMT and tumor aggressiveness and metastases [40]. As αvβ3 integrin facilitates the interactions with the TME and angiogenesis facilitates the immune evasion, β1 plays a role in ECM remodeling and resistance to chemotherapies for TNBC [41]. In addition to altering integrin expression, integrin dysregulation also alters the tumor microenvironment by stiffening the matrix and recruiting stromal components to promote tumor growth and dissemination [42, 43]. The aberrant integrin activities form a prometastatic milieu and represent attractive therapeutic targets in breast cancer [44]. Monoclonal antibodies, small molecule inhibitors, and other anti-integrin therapies promise to counteract their contribution to breast cancer progression [45].

## Mechanisms driving breast cancer metastasis

### Integrins and TGF-β in tumor microenvironment

Integrins and TGF-β are major mediators of the TME in breast cancer metastasis. αvβ6 and αvβ8 integrins activate latent TGF-β stored in the ECM to increase TGF-β signaling and trigger EMT, immune evasion, angiogenesis and ECM remodeling. The stimulation of specific integrin stages by TGF-β generates a positive feedback loop reinforcing tumor progression by upregulating integrin expression [46]. Integrin signaling also affects the TGF-β activation through the mechanical properties of the ECM, which enhance cytoskeletal remodeling, cell migration, and invasion [47]. Using high metastatic MDA-MB-231 cells as source cells, Costa et al. showed that ECM derived from these cells induces EMT in low metastatic MCF-7 cells through integrin αvβ3 signaling and crosstalk of TGFβ receptors. Kistrin (blocking integrin signaling, EMT) suppressed this effect, identifying integrin pathways as potential antimetastatic targets [48]. Integrin-TGF-β interactions extend to stromal cell activity, where cancer-associated fibroblasts (CAFs) play a pivotal role [49]. CAFs secrete TGF-β and cytokines like IL-6, which amplify integrin activation and promote EMT by upregulating transcription factors such as Snail and ZEB [50]. Mori et al. showed that fibroblast growth factor 1 (FGF1) amplifies TGF-β1-induced EMT in mammary epithelial cells through integrin αvβ3 and FGFR1 crosstalk. Inhibiting β3 integrin or FGFR1 effectively suppressed this synergy, demonstrating the potential of combined targeting strategies [51]. Similarly, Katoh et al. revealed that CAF-secreted tenascin C (TNC) enhances EMT by interacting with integrins αvβ6 and αvβ1, promoting FAK phosphorylation. Neutralizing these integrins blocked EMT and tumor progression, emphasizing their therapeutic relevance [52].

Integrin-TGF-β interactions extend to stromal cell activity, where cancer-associated fibroblasts (CAFs) play a pivotal role [53]. CAFs secrete TGF-β, IL-6, and ECM proteins, activating signaling pathways like Smad and integrin signaling. These pathways enhance Snail, Twist, and ZEB, promoting cell plasticity, invasion, and therapy resistance [54]. CAFs also remodel the ECM, increasing its stiffness and amplifying EMT signals, thereby facilitating tumor progression and metastasis [55]. Wen et al. identified CAF-derived IL32 as a key driver of breast cancer metastasis via integrin β3–p38 MAPK signaling. IL32 binds to integrin β3 on tumor cells, inducing EMT markers (FN, CDH2, VIM) and enhancing invasion. Blocking integrin β3, IL32, or p38 MAPK signaling significantly suppressed EMT and metastasis, highlighting the IL32-integrin β3 axis as a promising therapeutic target for CAF-induced tumor progression​ [56]. Chronic inflammation further exacerbates EMT through integrin-TGF-β crosstalk. Pro-inflammatory cytokines like TNF-α and IL-6 enhance integrin signaling, which in turn amplifies TGF-β signaling [57]. Liao et al. demonstrated that TNF-α and TGF-β1 co-stimulation enhances TAK1 activation, sustaining Smad2/3, MAPK, and NF-κB signaling. This feedback loop drives tumor progression and metastasis. Additionally, it was identified that CAF-derived IL32 as a driver of inflammation-induced EMT through integrin β3 and p38 MAPK signaling, suggesting that targeting this axis could mitigate inflammation-driven tumor progression​ [58]. Integrin-mediated feedback mechanisms are also critical to therapy resistance in breast cancer [59]. ECM stiffness sustains TGF-β signaling through integrin activation, promoting tumor cell plasticity, invasion, and resistance to treatment [60]. Kiefel et al. reported that TGF-β1-induced EMT upregulates L1CAM, which activates NF-κB signaling via integrins and enhances tumor motility and invasion [61]. Similarly, Yousafzai et al. highlighted Kindlin-2 as a stabilizer of β1-integrin-TβRI complexes, essential for oncogenic signaling in TNBC.

**Fig. 2:**
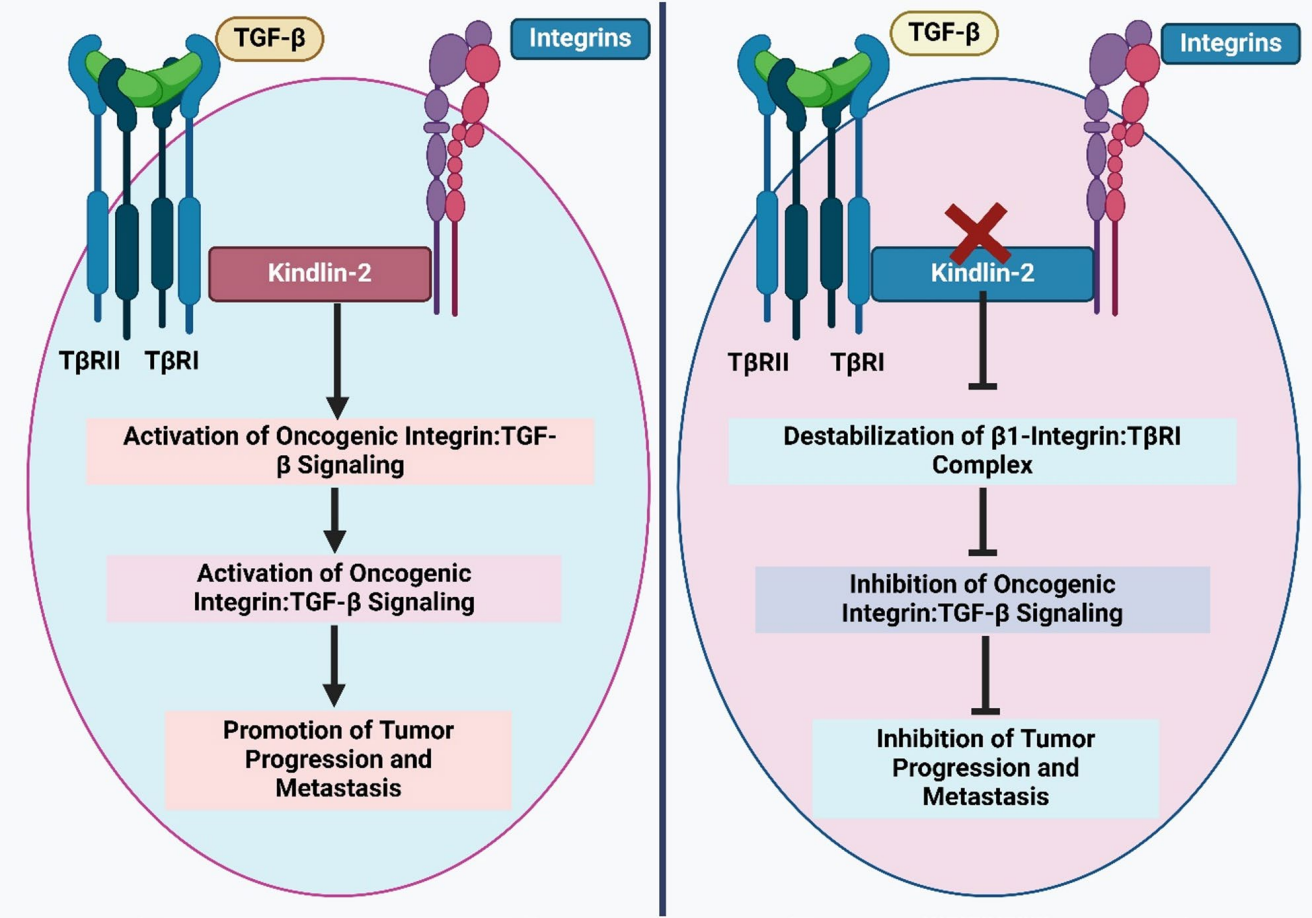
The figure illustrates the role of Kindlin-2 in the regulation of integrin-mediated TGF-β signaling and its implications for tumor progression and metastasis. On the left, Kindlin-2 promotes the stabilization of the β1-integrin: TβRI complex, leading to the activation of oncogenic integrin: TGF-β signaling. This activation drives tumor progression and metastasis. On the right, the absence or inhibition of Kindlin-2 results in the destabilization of the β1-integrin: TβRI complex, thereby inhibiting oncogenic integrin: TGF-β signaling

Disrupting this complex inhibited downstream pathways, tumor growth, and metastasis, offering a promising therapeutic approach for TNBC​ [62]. Therapeutic targeting of the integrin-TGF-β axis offers a promising approach to disrupting EMT and tumor progression [63]. Strategies include blocking integrin activation using inhibitors or antibodies, reducing ECM stiffness, or interfering with TGF-β signaling via Smad inhibitors or TGF-β receptor blockers [64]. These interventions disrupt the feedback loop between integrins and TGF-β, reducing EMT transcription factor activity, tumor invasiveness, and metastasis while enhancing therapeutic sensitivity [65]. Yousafzai et al. revealed Kindlin-2 as a stabilizer of β1-integrin, TβRI complexes, critical for oncogenic signaling in TNBC. Disrupting Kindlin-2 degraded β1-integrin and TβRI, inhibiting downstream pathways, tumor growth, and metastasis as shown in Fig. 2. Restoring Kindlin-2 re-established oncogenic activities, highlighting its role as a therapeutic target for TNBC treatment​ [62]. The integrin-TGF-β axis represents a critical convergence point for pathways driving EMT and metastasis in breast cancer. The evidence underscores the need for targeted therapies that disrupt this interplay to reduce tumor invasiveness, immune evasion, and resistance to existing treatments. Strategies such as integrin inhibitors or TGF-β pathway blockers hold promise in mitigating these effects and improving patient outcomes (Fig. 2).

### Integrin-TGF-β axis in breast cancer EMT

The integrin-TGF-β axis plays a pivotal role in driving EMT, a crucial process in breast cancer metastasis [66]. Integrins, such as αvβ6 and αvβ3, activate latent TGF-β, which induces EMT through both Smad-dependent and non-canonical pathways [67]. This activation suppresses epithelial markers like CDH1 and upregulates VIM and CDH2, enabling tumor cells to gain motility and invasiveness [68]. Integrin signaling, through pathways such as focal adhesion kinase (FAK) and PI3K/AKT, synergizes with TGF-β to remodel the cytoskeleton and disrupt cell-cell junctions [69]. This crosstalk establishes a pro-metastatic phenotype, making it a significant therapeutic target in metastatic breast cancer [70].

Several mechanistic investigations have shown that integrins as well as TGF-β are not parallel and independent pathways but tightly interacting signalling modules. αv-containing integrins (αvβ6 and αvβ8) recognise the LAP of TGF-β and convert latent complexes into active TGF-β by means of mechanical strain or by means of proteolytic process to form a direct pathway through which ECM engagement reinforces TGF-β signaling and EMT [34, 46, 67]. In mammary epithelial cells, β3 integrin is integrated with TβR-II and Src, where Tyr284 phosphorylation of TβR-II, Grb2 recruitment, and p38 MAPK activation occur and then inhibiting β3 integrin provides the return effect of cytostatic activity of TGF-β and blocks TGF-β induced oncogenic EMT and invasion just as an example of integrin-TGF-β receptor crosstalk [71–73]. On the other hand, TGF-β signalling may remodel integrin assembly and utilisation: Parvani et al. demonstrated that TGF-β-elicited β1 integrin to β3 integrin switching is necessary to maintain EMT, motility, and metastasis [74], whereas Yousafzai et al. found Kindlin-2 to assemble β1-integrin-TβRI complexes and preserve TGF-β-oncogenic signaling in triple-negative breast cancer [62]. The tumour microenvironment is also experiences cross-talk. Highly metastatic breast cancer cells depositing ECM activate EMT in otherwise low-metastatic cells by αv-integrins-binding TGF-β receptors, and tenascin C and IL-32 secreted by CAFs promote EMT through p38 MAPK/TGF-β and/or Smad pathway co-stimulation [48, 51, 52, 56]. These studies, in combination, indicate a model wherein integrin not only positively regulates and spatially clusters TGF-β receptors at the cell surface, but is reciprocally regulated by TGF-β, forming a positive feedback loop that promotes EMT, invasion, and metastatic outgrowth in breast cancer.

The dual role of β1 and β3 integrins in TGF-β-induced EMT and metastasis highlights the complexity of this axis. Parvani et al. demonstrated that inactivating β1 integrin impairs TGF-β-driven motility in normal and malignant MECs. However, β3 integrin compensates in malignant MECs, restoring EMT phenotypes, tumor growth, and metastasis. This finding underscores the need for dual-targeting strategies against β1 and β3 integrins for effective anti-metastatic therapies​ [74]. WAVE3, an actin-binding protein, is critical for regulating cytoskeletal remodeling, cell morphology, and motility during EMT. Taylor et al. identified WAVE3 as a mediator of TGF-β-induced EMT in TNBC. TGF-β upregulates WAVE3 via Smad2- and β3 integrin-dependent mechanisms, driving EMT and metastasis. Depleting WAVE3 suppressed EMT, migration, and tumor growth, highlighting its therapeutic potential in countering TGF-β-stimulated metastasis [75].

The β6 integrin is another key mediator in EMT and cancer progression, functioning through its interaction with TGF-β to activate downstream transcription factors such as Snail and ZEB [76]. Li et al. demonstrated that blocking β6 integrin rescued EMT phenotypes and decreased COX-2 expression via PI3K/AKT dephosphorylation. These findings position β6 integrin as a promising treatment target in breast cancer​ [77]. αvβ3 integrin, highly expressed in aggressive tumors, mediates TGF-β-induced EMT and cooperates with ECM components and hypoxia to drive cancer progression. Kariya et al. demonstrated that αvβ3 integrin promotes partial EMT, characterized by mesenchymal traits and retained epithelial markers. This partial EMT was abolished by microRNA-200, which suppresses αvβ3 integrin expression, suggesting αvβ3 integrin as a target for novel therapeutic interventions [78]. Basal-like breast cancer, a highly aggressive subtype, exhibits a high expression of EMT regulators such as δEF1. At the same time, other EMT markers like Snail, Twist, and Slug show limited correlation with aggressiveness. Shirakihara et al. identified integrin α3 (ITGA3) as a marker of EMT induced by TGF-β and FGF-2 in basal-like cancers. Targeting ITGA3 through MEK 1/2 inhibition downregulated δEF1 and ITGA3, highlighting its possibility as a treatment target in aggressive EMT-driven cancers​ [79].

During mammary tumorigenesis, genetic and epigenetic alterations disrupt the cytostatic effects of TGF-β, enabling it to promote EMT, metastasis, and invasion [71]. Galliher et al. revealed that β3 integrin enhances TGF-β-induced EMT and invasion by interacting with TβR-II and promoting Src-mediated tyrosine phosphorylation of TβR-II. Blocking β3 integrin restored TGF-β’s tumor-suppressive functions and prevented MAPK activation, making β3 integrin a critical target in the progression of breast cancer [71]. Twist1, a key transcription factor in EMT, drives metastasis by regulating genes such as miRNA-10b, AKT2, and CDH1 [80]. In addition to transcription factors, multiple lncRNAs act as competing endogenous RNAs to modulate EMT-related pathways. RHPN1-AS1 facilitates tumorigenesis via miR-485-5p sponging and TOP2A derepression, showing the layered regulatory network controlling EMT [81]. Yang et al. demonstrated that Twist upregulates ITGB1 (β1 integrin), activating FAK, ILK, and PI3K/AKT pathways, which promote EMT and invasion. Knockdown of Twist or β1 integrin suppressed these processes, presenting the Twist-ITGB1 axis as a viable target for therapeutic intervention. The integrin-TGF-β axis operates at the intersection of numerous signaling networks, making it a critical driver of EMT and metastasis in breast cancer. Its modulation involves not only the direct interaction between integrins and TGF-β but also their influence on key transcription factors, cytoskeletal dynamics, and extracellular matrix remodeling. This intricate crosstalk emphasizes the need for targeted approaches that disrupt integrin-TGF-β signaling while addressing tumor heterogeneity and resistance mechanisms.

In the light of inherent vulnerability, most of the risk today measurable regarding the integrin-TGF-β axis is due to nuclear-coded variations, as opposed to mitochondrial heredity. Germline polymorphisms of TGF-β pathway genes, including the low-penetrance TGFBR1*6A allele and other *TGFB1/TGFBR1* variants, are linked to a mild (approximately 1.1-1.3-fold) risk of breast cancer and augment TGF-β-induced proliferation, migration, and ERK/Rho-GTP signalling and hence predisposes to EMT and metastatic behaviour [82, 83]. High- and moderate-penetrance genes including *BRCA1*,* BRCA2*,* PALB2*,* ATM*, and *CHEK2* also, with each other, contribute to a large fraction of hereditary breast cancer via nuclear mechanisms that overlap with the DNA damage responses, TGF-β signaling and the invasive phenotype [84, 85]. Contemporary reviews summarize evolving trends in breast cancer genetics, risk assessment, and screening strategies, reinforcing the clinical relevance of hereditary susceptibility in breast cancer management [86, 87]. In comparison, common polymorphisms in integrin genes (*ITGB3* and other integrin subunits) demonstrate in the best case weak or situation-specific relationships between breast cancer susceptibility [88]. Though a few genotypes have been implicated with incremented risk of metastasis, proposing that germline variability could operate by refining αvβ3 signaling in the metastatic environment must not be as a solid foremost contributor to predisposition [89].

Mitochondrial DNA (mtDNA) is maternally inherited and is not subjected to simple Mendelian segregation, but under the influence of mutations in mtDNA and mitochondrial dysfunction, TGF-β and integrin signaling may be amplified indirectly by increasing reactive oxygen species, metabolic reprogramming, and mitochondrial retrograde signalling [90, 91]. In breast cancer, MAEL has been demonstrated to facilitate metabolic reprogramming and cancer growth by promoting chaperone-mediated autophagy-dependent degradation of key metabolic enzymes [92]. In models of breast and other epithelial cancers, experimental depletion, or damage of mtDNA has been shown to cause EMT and upregulation of TGF-β/Smad/SNAIL pathways, upregulation of integrin expression and maximization of migration ability [93]. Mechanotransduction (αvβ3-, β5-mediated signaling) can, in its turn, remodel mitochondrial bioenergetics and induce a glycolytic, migration-competent phenotype, further connection ECM cue, mitochondria and the EMT [94, 95].

Emerging literature also corelates EMT and TGF-β signaling with ferroptosis regulation via ferritinophagy-mediated iron metabolism. For example, COPZ1 has been demonstrated to modulate ferroptosis via NCOA4-mediated ferritinophagy [96],, highlighting an additional redox-sensitive vulnerability in cancer growth. Currently, though, there exist no robust epidemiological data to enable a significant calculation of the percentage contribution of nuclear versus mitochondrial inheritance to the specific malfunctioning of αvβ3 integrin-TGF-β receptors, in particular. Heritable predisposition should therefore be considered polygenic and obviously nuclear-influenced, where the change of the mitochondrial dysfunction and the ability of the mitochondrial DNA to alter the provisional action of EMT and metastatic fitness, but not necessarily being quantitatively significant, are modulators of the predisposition.

### Invasion and colonization

Invasion and colonization represent critical steps in breast cancer metastasis, orchestrated through the interplay of integrins and TGF-β signaling [62]. Integrins such as αvβ3 and β1 facilitate tumor cell migration by promoting cytoskeletal reorganization and ECM degradation, primarily mediated by MMPs [97]. Concurrently, TGF-β signaling amplifies these effects by inducing EMT, enabling cancer cells to enter neighboring tissues, enter circulation, and disseminate to distant sites. Upon reaching secondary organs, integrins support extravasation and colonization by enhancing cell survival and adhesion in foreign microenvironments, underscoring the importance of these signaling networks as therapeutic targets in metastatic breast cancer [98]. β5 integrin plays an essential role in breast cancer invasion by mediating cell adhesion, migration, and survival through interactions with ECM components like vitronectin. Bianchi et al. demonstrated that β5 integrin amplifies TGF-β signaling, promoting EMT and enhancing tumor invasiveness and therapy resistance. Depleting β5 integrin impaired cell-matrix adhesion and reduced integrin signaling despite no changes in CDH1 expression. These findings suggest that β5 integrin is a promising target for disrupting EMT and tumor progression​ [72]. Integrins containing the β1 subunit are central to invasion and colonization, providing cancer cells with physical and chemical signals that drive survival and proliferation [99]. Preclinical studies have shown that β1 integrin antagonists inhibit tumor growth and enhance sensitivity to chemotherapy and irradiation [100]. However, Truong et al. highlighted a paradoxical role for β1 integrins, showing that their loss activates TGF-β signaling, disrupts the ZEB2/miR-200 balance, and promotes single-cell migration in TNBC. These findings caution against β1 integrin-targeting therapies without careful consideration of tumor context​ [101]. α2β1 integrin, while implicated in multiple cancers, exhibits a dual role in breast cancer progression, particularly in bone metastases [102]. Moritz et al. found that α2β1 integrin expression correlates inversely with bone-metastatic potential. Overexpression of α2 integrin enhanced primary tumor growth and dissemination to bone, yet once in the bone environment, tumors showed reduced α2β1 expression and increased osteolytic signaling​ [103]. These findings underscore the complexity of targeting α2β1 integrin, as its inhibition may limit primary tumor growth but exacerbate bone metastases.

**Fig. 3:**
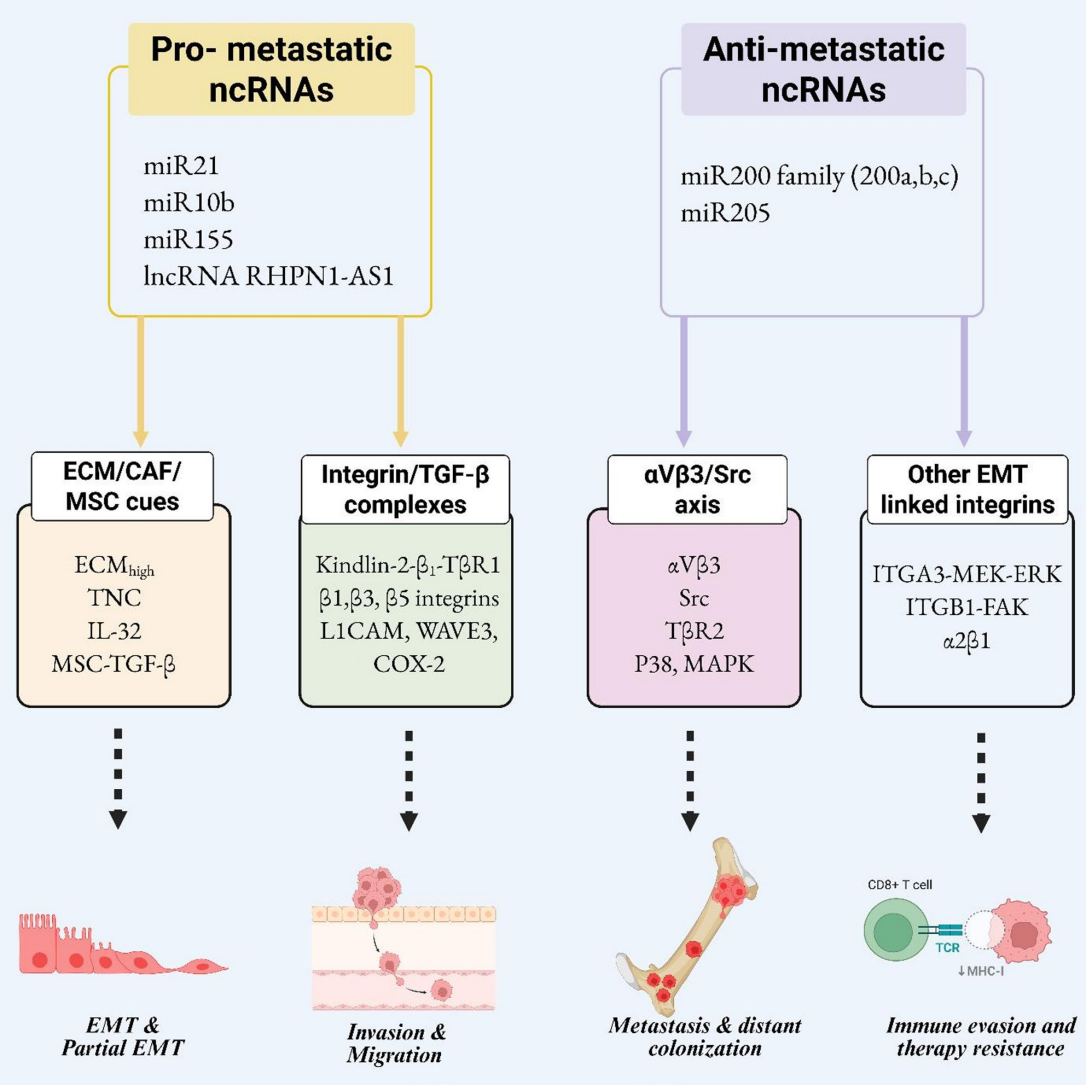
The figure illustrates the ncRNA-mediated control of integrin–TGF-β signaling in breast cancer metastasis. It depicts how pro-metastatic ncRNAs converge on ECM/CAF/MSC-derived cues and integrin–TGF-β complexes to amplify EMT, invasion, and migration. In contrast, anti-metastatic ncRNAs are shown as negative regulators that counteract αvβ3/Src signaling and other EMT-linked integrins, thereby restraining metastatic spread. Together, these ncRNA networks fine-tune key integrin modules, including Kindlin-2–β1–TβRI complexes, αvβ3/Src/p38 MAPK signaling, and ITGA3–MEK–ERK or ITGB1–FAK axes, which collectively shape EMT status, migration, distant colonization, and immune evasion

The TME is further shaped by local adipose tissues recruited from bone marrow and MSCs in response to tumor-secreted factors [104]. MSCs promote breast cancer invasion and colonization by differentiating into CAFs, secreting angiogenic factors, and supporting cancer stem cell properties [105]. McAndrews et al. revealed that MSC-derived TGF-β enhances the directional migration of breast cancer cells by increasing traction forces, elongation, and MMP activity. These findings suggest MSC-derived TGF-β as a potential therapeutic target to inhibit metastasis​ [106]. The dual role of TGF-β as a tumor suppressor and promoter is evident during breast cancer progression. While TGF-β suppresses tumorigenesis through Smad2/3-mediated cell cycle arrest, it drives metastasis by activating pathways involving β1 integrin, RhoA, MAPK, and PI3K [107, 108]. Galliher et al. identified the αvβ3 integrin/Src/Y284/TβR-II axis as a critical driver of TGF-β-induced oncogenic signaling. This axis promotes EMT, invasion, and MAPK activation while blocking αvβ3 integrin restored TGF-β’s cytostatic function, positioning this pathway as a potential therapeutic target​ [109]. The integrin-TGF-β axis facilitates a cooperative signaling network that drives invasion and colonization, critical steps in breast cancer metastasis. Therapeutic approaches targeting integrins (e.g., β5, β1, and αvβ3) or TGF-β signaling hold promise for disrupting these processes. However, the context-dependent roles of these molecules necessitate precise strategies to avoid unintended consequences, such as enhanced metastatic potential or therapy resistance (Table 1). Also, the ncRNA-mediated control of integrin–TGF-β signaling in breast cancer metastasis were depicted in Fig. 3.

**Table 1:** This table summarizes studies on integrins and TGF-β in breast cancer metastasis mechanisms

Findings	Mechanism	Key proteins involved	Metastasis activators	Reference
ECM from high-metastatic cells induces EMT	Integrin αvβ3, TGF-β receptor interaction	Integrin αvβ3, TGF-β	Integrin αvβ3, TGF-β	[48]
FGF1 amplifies TGF-β1-induced EMT through integrin	Integrin αvβ3, FGFR1 signaling	Integrin αvβ3, FGFR1	Integrin αvβ3, FGFR1	[51]
Integrins αvβ6 and αvβ1 mediate TNC-induced EMT	Integrins αvβ6, TGF-β1 signaling	Integrins αvβ6, αvβ1	Integrins αvβ6, TNC	[52]
CAF-derived IL32 drives metastasis via integrin β3	IL32-integrin β3 signaling	IL32, Integrin β3	IL32, Integrin β3	[56]
TNF-α and TGF-β1 co-stimulation drives EMT.	TGF-β1, TNF-α synergy signaling	Smad2/3, NF-κB	TGF-β1, TNF-α	[55]
TGF-β1-induced EMT upregulates L1CAM expression	L1CAM-integrin crosstalk signaling	L1CAM, Integrins	L1CAM, Integrins	[61]
Kindlin-2 stabilizes β1-Integrin: TβRI complexes	Kindlin-2-mediated β1-Integrin stabilization	Kindlin-2, β1-Integrin	Kindlin-2, β1-Integrin	[62]
β1 and β3 integrins drive TGF-β-induced EMT	β1, β3 integrin-dependent EMT signaling	β1, β3 Integrins	β1, β3 Integrins	[74]
WAVE3 mediates TGF-β-induced EMT in TNBC	Smad2, β3 integrin regulates WAVE3	Smad2, β3 Integrins	WAVE3, TGF-β	[76]
β6 integrin mediates TGF-β1-induced EMT	β6 integrin regulates COX-2, PI3K/Akt	β6 Integrin, COX-2	β6 Integrin, TGF-β1	[77]
αvβ3 integrin enhances EMT via Src-TβR-II	αvβ3 integrin activates Src, TβR-II	αvβ3 Integrin, Src	αvβ3 Integrin, Src	[71]
αvβ3 integrin drives partial EMT and invasion	αvβ3 integrin promotes migration, EMT	αvβ3 Integrin	αvβ3 Integrin	[78]
ITGA3 enhances EMT in breast cancer cells	ITGA3 regulates MEK-ERK signaling	ITGA3, MEK-ERK	ITGA3	[79]
Twist-ITGB1-FAK axis drives EMT.	Twist upregulates ITGB1, activates FAK	Twist, ITGB1, FAK	Twist, ITGB1	[100]
β5-integrin drives TGF-β-induced EMT	β5 integrin regulates Smad signaling	β5 Integrin, Smad	β5 Integrin, TGF-β	[105]
β1 integrins suppress migration in TNBC	β1 integrins maintain epithelial phenotype	β1 Integrins	β1 Integrins	[101]
MSCs promote breast cancer migration via TGF-β	MSCs secrete TGF-β, activate Rho	TGF-β, Rho	TGF-β, MSCs	[106]
αvβ3 integrin activates Src, promotes EMT	Src phosphorylates TβR-II, MAPK activation	αvβ3 Integrin, Src	αvβ3 Integrin, Src	[109]
α2β1 integrin correlates with bone metastasis	α2β1 integrin regulates osteolytic signaling	α2β1 Integrin	α2β1 Integrin	[107]

## Therapeutic implications

### Targeting integrins

Targeting integrins represents a promising therapeutic strategy for mitigating breast cancer progression and metastasis [110]. These cell-surface adhesion receptors regulate interactions between tumor cells and the ECM, facilitating processes such as cell adhesion, migration, and invasion [111]. Monoclonal antibodies (e.g., against αvβ6 and αvβ3) and small molecule inhibitors (e.g., GLPG0187) have shown efficacy in preclinical models by disrupting integrin-mediated signaling, ECM interactions, and TGF-β activation [112]. These strategies inhibit tumor cell motility, angiogenesis, and immune evasion, highlighting their potential to improve treatment outcomes in breast cancer [113]. The integrin-TGF-β axis is particularly critical in driving EMT and metastatic signaling. Desai et al. revealed that integrin β6 synergizes with growth factor receptors such as HER2 and EGFR to mediate EMT and migration in luminal breast cancers. This EMT process, marked by elevated αvβ6 expression and VIM upregulation, correlates with poor disease-free survival. Inhibition of TGF-β signaling using lapatinib reverses EMT by reducing SNAI1, SNAI2, integrin β6, and MMP-9 expression, demonstrating the therapeutic potential of integrin targeting in aggressive luminal tumors [114].

In TNBC, integrins play an important role in immune resistance and therapy resistance. Bagati et al. identified the αvβ6-TGF-β-SOX4 axis as a key driver of immune evasion, with SOX4 suppressing tumor-intrinsic immune pathways. Blocking αvβ6 with monoclonal antibodies downregulated SOX4 expression and enhanced the efficacy of immunotherapy, offering a novel approach for resistant TNBC subtypes [115]. Similarly, Smiraglia et al. demonstrated that integrin β5 mediates angiogenesis and tumor progression in TNBC through Src-FAK and MEK-ERK signaling. Depleting β5 integrin reduced VEGF-A expression, migration, and proliferation, positioning β5 as a valuable target for treating aggressive cancers​ [116, 117]. Integrins are also implicated in metastatic colonization [118]. Wendt et al. highlighted Pyk2 as a critical mediator of TGF-β-driven EMT and metastatic outgrowth in breast cancer. Pyk2 stabilizes β1 integrin expression and supports secondary tumor growth in pulmonary microenvironments. Inhibiting Pyk2 blocks metastatic colonization, emphasizing its therapeutic potential in managing breast cancer dissemination​ [119]. The αv family of integrins (e.g., αvβ5, αvβ3, αvβ6) has a central role in tumor progression, interacting extensively with TGF-β signaling to amplify oncogenic responses. Beckley et al. identified the αvβ3 integrin: Src: TβR-II: Grb2:p38 MAPK axis as a critical pathway in TGF-β-driven EMT and invasion. Inhibiting Grb2 or TβR-II phosphorylation impaired tumor growth and metastasis, highlighting this axis as a therapeutic target​ [73].

Similarly, Li et al. demonstrated that targeting αv integrin with GLPG0187 inhibits migration and metastasis in breast cancer models. The combination of GLPG0187 with zoledronate and paclitaxel further enhanced efficacy, suggesting αv integrin as a promising target for integrin-overexpressing tumors​ [120]. Grb2 further exemplifies the interplay between integrins and oncogenic signaling [121]. As an adaptor protein linking RTKs to the RAS-MAPK pathway, Grb2 shows a crucial role in TGF-β-mediated EMT and metastasis [122]. Beckley et al. showed that Grb2 binds phosphorylated TβR-II (Y284), activating p38 MAPK independently of Smad3 signaling. Disrupting this pathway impaired tumor growth and metastasis, providing another avenue for integrin-targeted therapies​ [73]. Overall, integrins and their associated signaling pathways, including αvβ6, β5, and β1 integrins, represent critical mediators of breast cancer progression. Targeting these integrins, either alone or in combination with other therapies such as immunotherapy, TGF-β inhibitors, or chemotherapy, holds substantial promise for reducing metastasis, improving therapy response, and enhancing patient outcomes.

Observable inconsistencies between the studies of single integrin subunits are, most likely tumour subtype-contextual, ECM-specific, and integrin switching (β1 to β3) during therapy. There are also discrete patterns of cross talk with TGF-β receptor, which further diversifies the results. These disparities will have to be resolved with the help of isoform-selective inhibitors and biomarker-stratified models and trials.

### Inhibiting TGF-β pathway

The TGF-β pathway is a pivotal regulator of EMT, immune evasion, and invasion, making it a prime therapeutic target in breast cancer [123]. However, the dual role of TGF-β necessitates selective inhibition of its pro-tumorigenic signaling pathways to minimize adverse effects [124]. Small molecule inhibitors targeting TGF-β receptors and Smad signaling have demonstrated promise in preclinical models by effectively blocking EMT and associated metastatic processes. TGF-β-driven EMT has been linked to resistance in hormone receptor-positive breast cancer. Tian et al. demonstrated that TGF-β-induced EMT enhances cytoplasmic accumulation of ER-α in luminal breast cancer cells, promoting tamoxifen resistance. EMT-induced upregulation of EGFR, IGF1R, and Src forms complexes with ER-α, activating MAPK signaling and driving anti-estrogen resistance. Targeting TGF-β, EGFR, IGF1R, and MAPK pathways restored tamoxifen sensitivity, highlighting EMT-mediated nongenomic ER-α signaling as a critical driver of therapy resistance​ [125]. Inhibitors of TGF-β signaling have shown efficacy in blocking EMT and associated downstream effects [126]. Research studied TGF-β-induced EMT using cell lines and identified small molecule inhibitors, such as GW788388 and SB431542, that block Smad phosphorylation and EMT. These inhibitors provide a foundation for developing novel strategies to disrupt TGF-β-driven tumor progression [127]​.

The interplay between p53 and TGF-β signaling further underscores the complexity of targeting this pathway [128]. Lam et al. revealed that pharmacological activation of p53 suppresses TGF-β3-induced EMT, migration, and invasion by downregulating targets like MMP2, MMP9, and integrin β3. p53 also inhibits EPHB2, a novel TGF-β3 target involved in tumor invasion. These findings highlight the antagonistic relationship between p53 and TGF-β3, presenting p53 activation as a potential therapeutic strategy [129]. TGF-β isoforms, particularly TGF-β2, play distinct roles in aggressive breast cancer subtypes. Kim et al. identified TGF-β2 as a driver of metastasis in TNBC, promoting upregulation of FN, MMP-2, and MMP-9. The natural compound silibinin suppressed TGF-β2 expression, reducing FN and MMP levels, cell migration, and lung metastasis. This positions silibinin as a potential therapeutic agent for targeting TGF-β2-mediated mechanisms in TNBC​ [130]. The inhibition of TGF-β signaling offers multiple therapeutic avenues for combating breast cancer progression. However, given the pathway’s duality, precision in targeting its oncogenic components while preserving tumor-suppressive functions is critical. Combining TGF-β inhibitors with therapies targeting related pathways, such as ER-α signaling or matrix-degrading enzymes, may further enhance efficacy and reduce metastatic potential.

Regarding experimental and translational aspects, there are a number of practical issues that must be taken into account when the TGF-β pathway inhibitors and integrin-targeted agents are used. Imaging-based biomarkers are increasingly employed to capture TME characteristics. PET/CT metabolic parameters have been correlated with tumor-infiltrating lymphocyte levels and survival outcomes in breast cancer [131], offering a non-invasive window into immune-metabolic interactions of the TME. Small-molecule ALK5/TGF-β receptor inhibitors, including SB431542 and GW788388, are generally used in vitro, usually on an epithelial or breast cancer cell line, at low concentrations of approximately 30–60 min of pre-treatment period prior to exogenous stimulation by TGF-β to allow effective prevention of Smad2/3 phosphorylation. In this case, SB431542 consistently inhibits EMT, migration, and invasion forced by TGF-β, as well as VEGF secretion in several different tumor models, whereas GW788388 and other ALK5 inhibitors decrease EMT and fibrotic markers and inhibit invasive growth in vitro and in vivo [132–134]. On the organism level, anti-fibrotic and anti-tumor effects of ALK5 inhibitors like GW788388 and galunisertib have demonstrated strong results in preclinical models, in that they improve the cardiac performance and reduce fibrosis or tumor burden with acceptable toxicity at optimal doses [134, 135]. Nevertheless, blanket inhibition of TGF-β signalling especially dual isoform targeting (TGF-β2/3) has demonstrated great cardiovascular and systemic-wide organ toxicity in animals, and certain ALK5 antagonist chemotypes have been linked to heart valve lesions, highlighting the importance of appropriate dose selection and isoform selectivity [136, 137].

Eligibility criteria in early-stage clinical trials of TGF-β pathway inhibitors (galunisertib, PF-06952229) and integrin-targeted (αvβ3- or pan-αv-blocking antibodies, and small molecules) agents, the patients are usually enrolled on the very high baseline eligibility criteria, such as good cardiac, hepatic, and renal function, baseline electrocardiography and echocardiography, and the absence of uncontrolled cardiovascular diseases [138–141]. Integrin antagonists including cilengitide, etaracizumabm and GLPG0187 have demonstrated, largely, good safety and tolerability (fatigue, nausea, thrombocytopenia, and mild bleeding being the most common adverse reactions) although they have produced modest responses in some cases and no reliable benefit to survival in solid tumors, despite producing a high potency in vitro (a gap between high anti-EMT potency in vitro and low clinical efficacy) [112–142]. In case of TGF-β inhibitors, intense cardiac monitoring of galunisertib trials has not demonstrated clinically significant cardiotoxicity, however, overall clinical benefits across cancers have so far been insignificant and far more context-specific [138, 139]. Due to the heterogeneity of experimental modalities, to tumour types and dose schedules, a single percent success cannot be attributed to these agents either at cell-culture level or full organism level, rather the available information indicate strong percent success of repression of EMT and invasion in cell-culture, plus inconsistent and model-dependent decreases in tumour volume and metastasis in patients, where patient clinical responses are yet to be revealed [132, 143, 144]. According to existing evidence, we expect that the best opportunities of clinical success would involve biomarker-guided combination procedures [145] (using ALK5/TGF-β accompanied by chemotherapy, anti-HER2 or immunotherapy in EMT- or TGF-β high tumours), along with cautious cardiac and organ monitoring and prevention of excessive blockade of TGF-β isoforms to restrict toxicity [135, 144].

### Combination therapies

Combination therapies that target both integrins and the TGF-β pathway offer synergistic potential to combat breast cancer metastasis [146]. Integrin antagonists disrupt ECM interactions and TGF-β activation, while TGF-β inhibitors suppress EMT, invasion, and immune evasion. When combined with chemotherapy or immune checkpoint inhibitors, these therapies target multiple hallmarks of cancer progression, improving efficacy, reducing resistance, and offering a comprehensive approach to aggressive and metastatic breast cancer. The interplay between integrins and the TGF-β pathway is exemplified by studies showing their cooperative role in metastasis suppression. Naber et al. demonstrated that BMP-7 inhibits TGF-β-induced invasion in metastatic breast cancer cells by suppressing integrin αvβ3 expression. While targeting β3 integrin reduced invasion, its overexpression counteracted BMP-7’s effects, positioning BMP-7 as a potential anti-invasive agent in combination with integrin inhibition​ [147]. Radiation therapy, while effective against primary tumors, can induce EMT and enhance metastatic potential. Rajput et al. found that thymoquinone, a bioactive compound, reverses radiation-induced EMT in breast cancer by restoring TGF-β balance. Thymoquinone pre-sensitization reduced mesenchymal marker expression (e.g., integrin αv, MMP-9, MMP-2) and increased epithelial markers, while paclitaxel-induced apoptosis in sensitized cells. This dual approach highlights the potential of combining natural compounds with chemotherapy to counteract therapy-induced metastasis ​ [148]. Lymphatic metastasis, a common route for tumor dissemination, is driven by integrin and TGF-β signaling. Salvo et al. showed that TGF-β-induced β3 integrin expression enhances tumor cell adhesion to lymphatic endothelium [149], promoting lymph node metastasis.

**Fig. 4:**
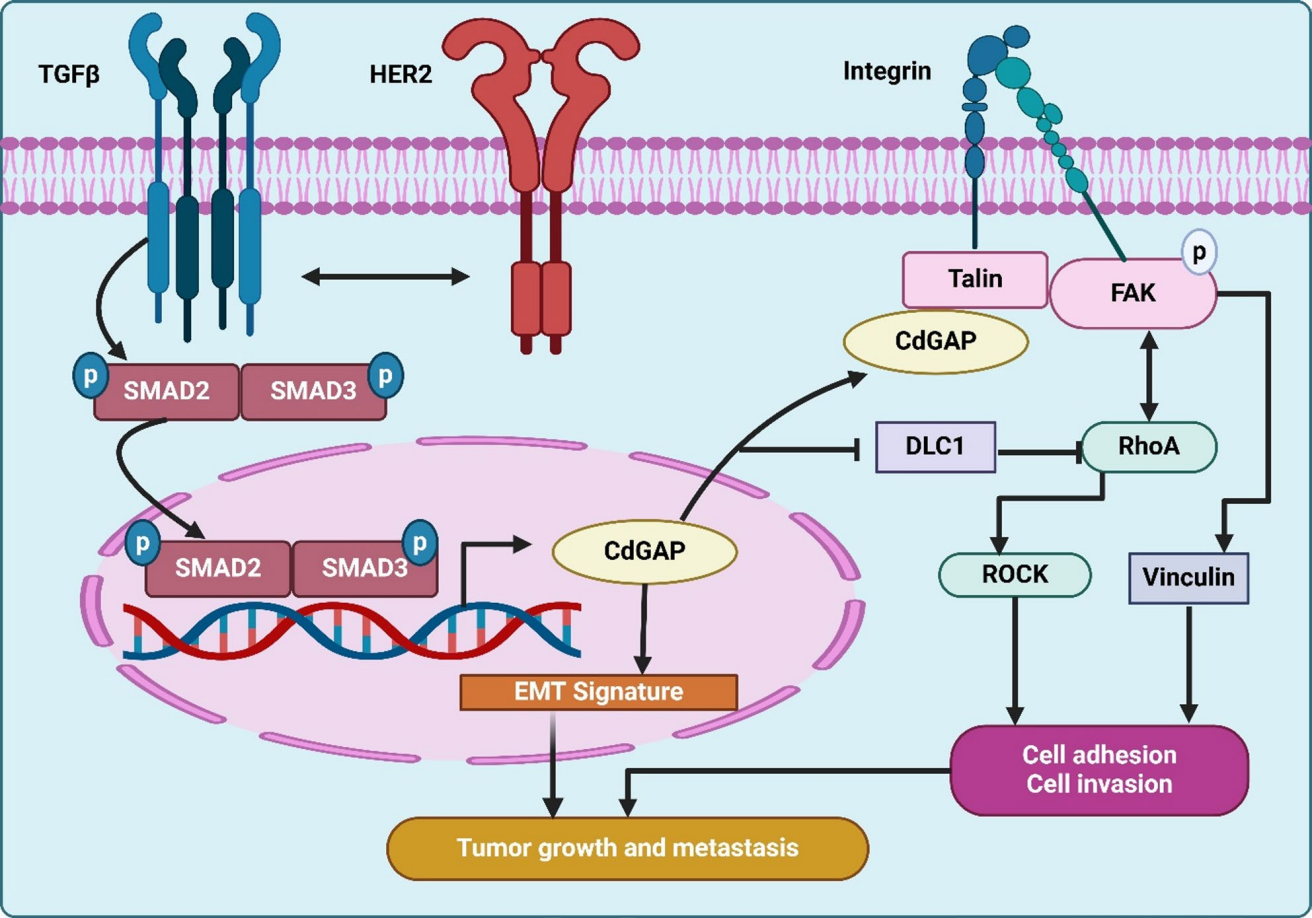
The figure illustrates the interaction between TGF-β signaling, HER2 signaling, and integrin pathways in promoting EMT, tumor growth, and metastasis. TGF-β activates Smad2 and Smad3 via phosphorylation, leading to their nuclear translocation and regulation of EMT-related gene expression, establishing an EMT signature. HER2 signaling interacts with TGF-β signaling to amplify the EMT response. Simultaneously, integrin signaling through Talin and FAK (Focal Adhesion Kinase) activates downstream pathways, including RhoA, ROCK, and Vinculin, which promote cell adhesion and invasion. CdGAP acts as a central mediator linking TGF-β/Smad and integrin/FAK signaling, contributing to EMT and tumor progression. DLC1 (Deleted in Liver Cancer 1) inhibits RhoA signaling, serving as a potential suppressor in this pathway

Dual therapy targeting TGF-β and β3 integrin significantly reduced metastasis in models refractory to TGF-β monotherapy, emphasizing the value of integrin-TGF-β combination strategies in reducing metastatic burden​. The synergy between TGF-β-induced EMT and EGFR signaling is a key driver of metastatic behaviors in aggressive breast cancer. Wendt et al. demonstrated that EMT enhances EGFR-driven invasion by disrupting EGFR- CDH1 interactions and forming oncogenic EGFR-TβR-II complexes. Post-EMT cells exhibited hyperinvasive responses to EGF, which were mitigated by focal adhesion kinase (FAK) inhibition. Targeting FAK effectively reversed EMT-induced aggression, offering a strategy to counteract the cooperative effects of TGF-β and EGFR on metastasis​ [150]. Emerging evidence underscores the role of Cdc42 GTPase-activating protein (CdGAP) in HER2 + breast cancer metastasis. He et al. identified CdGAP as a driver of EMT and metastasis via TGF-β signaling, where it modulates focal adhesion dynamics through interactions with talin. Knockdown of CdGAP in HER2-transformed cells reduced tumor motility, invasion, and metastasis, as shown in Fig. 4. CdGAP’s high expression correlates with lymph node invasion and poor disease-free survival, positioning it as a promising therapeutic target in HER2 + metastatic breast cancer​ [151]. Combination therapies that integrate integrin inhibition with TGF-β pathway targeting demonstrate substantial promise in reducing metastatic potential and improving therapeutic response. Strategies such as BMP-7 administration, natural compounds like thymoquinone, and targeted therapies against integrin β3 and CdGAP offer multi-faceted approaches to addressing breast cancer progression (Fig. 4).

Beyond tumor-intrinsic effects, cancer-host communication contributes to treatment toxicity. Small extracellular vesicles derived from breast cancer cells have been implicated in anthracycline-induced cardiotoxicity through ferroptosis in cardiomyocytes, underscoring the systemic consequences of tumor-derived signaling [152]. There are a large number of integrin subunits and TGF-β pathway components which have been suggested as therapeutic targets, though the quality and strength of the supporting evidence differ significantly. A significant fraction of targets disclosed are based mainly on in-vitro findings of inhibiting EMT signatures or migration, yet only a small set of agents (e.g., αvβ3/αvβ6 antagonists, ALK5 inhibitors like galunisertib) show consistent effectiveness in animal models and have moved to early-phase clinical testing. The overall clinical responses have been relatively low with a high degree of context dependence attributable to heterogeneity of tumour subtypes, as well as redundancy in integrin/TGF-β biological pathways. Some other studies may present seemingly contradictory data among integrin subunits due to integrin switching, ECM context, and model-related variations, hence the importance of patient selection using biomarkers. Combined, the existing literature gives a good mechanistic explanation but still lacking clinical confirmation, which is to be replaced by isoform-selective inhibitors and combination therapy, as well as careful translational research to correlate inhibition of the targets with patient outcome.

## Conclusion and future perspective

The integrin-TGF-β axis is a major convergence point in the progression of breast cancer: from EMT to escape immunity and invasion to metastasis. Latent TGF-β is activated by integrin αvβ3, αvβ6 and β1 as activators and also enhances its signaling via Smad and non-Smad signaling pathways. Crosstalk with this enables cytoskeletal reorganization, ECM remodeling, and cell motility for a feedback loop to perpetuate tumor progression. Moreover, integrin subunits β5 and αvβ3 facilitate lymphatic and distant metastases through increasing adhesion to secondary sites, and α2β1 and β1 integrins are involved in bone metastasis and resistance to therapy. TGF-β′s dual role as both a tumor suppressor in the early stages and promoter in the later stages makes it even more difficult to target, emphasizing the complexity of this signaling network. Therapeutic strategies targeting the integrin-TGF-β axis have demonstrated significant preclinical promise. Monoclonal antibodies (e.g., against αvβ6 and β3), small molecule inhibitors (e.g., GLPG0187), and combination therapies with chemotherapeutic agents (e.g., paclitaxel and zoledronate) have shown potential to disrupt EMT, invasion, and metastasis. Moreover, agents like BMP-7 and natural compounds such as thymoquinone illustrate innovative approaches to counteracting TGF-β-mediated aggression. Despite these advancements, tumor heterogeneity, context-dependent integrin roles, and the plasticity of EMT states remain formidable challenges.

However, integrative targeting of therapeutic agents with precision medicine will be key to unlocking the therapeutic potential of this axis. The next steps are crucial to identify robust biomarkers to stratify patients, selectively inhibit pro-tumorigenic pathways, and overcome resistance mechanisms. In addition, therapies designed to block integrins and TGF-β, as well as related signaling pathways (e.g., EGFR and FAK), may effectively complement one another in inhibiting metastatic progression. Future research should address how, in different microenvironments, integrins and TGFβ separately or in concert play unique roles in various aspects of tumor progression, develop combinatorial treatment strategies based on their identified interactions with the TME, and determine patient selection based on biomarkers that validate the roles in the clinical setting. It will enable these findings to be translated into actions, which will produce a significant reduction in the outcomes for patients with metastatic breast cancer.

## Data Availability

No datasets were generated or analysed during the current study.

## Declarations

### Generative AI and AI-assisted technologies in the writing process

During the preparation of this work, the authors used Generative AI and AI-assisted tools solely to improve the language, grammar, and readability of the manuscript. No AI tools were used for generating substantive content, data analysis, interpretation of results, or drawing scientific conclusions. We have used Turnitin for the plagiarism check of our manuscript. All intellectual contributions, study design, data collection, analysis, and final interpretations were conceived and executed by the authors. The authors reviewed and verified the accuracy and appropriateness of all AI-assisted edits and take full responsibility for the content of this publication

## Abbreviations

AKT: Protein kinase B
ALK5: Activin receptor-like kinase 5
BMP-7: Bone morphogenetic protein 7
BRCA1/2: Breast cancer type 1/2 susceptibility protein
CAF/CAFs: Cancer-associated fibroblast(s)
CDH1: E-cadherin
CDH2: N-cadherin
CT: Computed tomography
ECM: Extracellular matrix
EGFR: Epidermal growth factor receptor
EMT: Epithelial-to-mesenchymal transition
ER-α: Estrogen receptor alpha
ERK: Extracellular signal-regulated kinase
FAK: Focal adhesion kinase
FN: Fibronectin
HER2: Human epidermal growth factor receptor 2
IGF1R: Insulin-like growth factor 1 receptor
IL-6: Interleukin-6
ITGA3: Integrin subunit alpha 3
ITGB1/3: Integrin subunit beta 1/3
JNK: c-Jun N-terminal kinase
L1CAM: L1 cell adhesion molecule
MAPK: Mitogen-activated protein kinase
MMP-2/9: Matrix metalloproteinase-2/9
MSC/MSCs: Mesenchymal stem cell(s)
NF-κB: Nuclear factor kappa-light-chain-enhancer of activated B cells
PET: Positron emission tomography
PI3K: Phosphoinositide 3-kinase
Pyk2: Proline-rich tyrosine kinase 2
SB431542: ALK5/TGF-β receptor inhibitor SB431542
SOX4: SRY-box transcription factor 4
TβR-I/II: Transforming growth factor-β receptor I/II
TGF-β: Transforming growth factor-β
TGFBR1: Transforming growth factor-β receptor 1
TME: Tumor microenvironment
TNBC: Triple-negative breast cancer
TNF-α: Tumor necrosis factor-alpha
VEGF/VEGF-A: Vascular endothelial growth factor / A
VIM: Vimentin
